# Preserved Sunshine

**Published:** 1885-05

**Authors:** 


### HALL'S JOURNAL OF HEALTH

Vol. 32. **MAY, 1885.** No. 5.

### Preserved Sunshine.

Light and life are inseparable, that is, such was the generally received opinion many years ago, and in accordance with it, houses were built, liberally supplied with windows, and as liberally now—but go along any of the fashionable streets of New York, and you will find not less than three, and often six, distinct contrivances to keep out the sunshine and gladness. First, the Venetian shutter on the outside; Second, the close shutter on the inside; Third, the blind which is moved by rollers; then, Fourthly, there are the lace curtains; Fifth, the damask or other material.

In the same train come the exclusion of external air by means of double sash, and a variety of patent contrivances to keep any little stray whiff of air from entering at the bottom, sides and tops of doors and windows. At this rate, we will, in due time, dwindle into Lilliputs, if indeed we do not die off sooner, with all science and art, and leave the world to begin anew, from the few sons of the forest, who persisted in eschewing civilization. We lay it down as a health axiom—*The more out-door air and cheery sunshine a man can use, the longer he will live.* But the *Preserved Sunshine!* What about it? That very same sunshine which so lavishly beamed upon our continent with all its tropical fervor in the earlier ages of creation, what has become of it? A casual reader of the JOURNAL will exclaim, "What a fool of a question that is!" Let us leisurely inquire into it; but in doing so we must take it for granted that the reader knows something.

In Central America, where the sun shines with all its brilliancy and fierceness, vegetation is of fabulous growth, of a luxuriance almost incredible.

But how does a tree grow? Without light no wood is made in any vegetable growth; the woody fibre is formed from *carbonic acid gas* being absorbed by the leaves and through the bark of any growth. But light separates the two constituents which compose this carbonic acid gas, carbon and oxygen, and two different uses are made of it; the oxygen is liberated, thrown out and breathed by animals and men, while the carbon or "coal" goes to form the woody fibre of the plant, which presents a kind of ring, plainly seen in sawing through any tree, the number of rings indicating the age of the tree in years; some of these rings are broader, some narrower, indicating most probably the more or less sunshine of that year, for a plant will not grow as much in a cold summer as in a warm one. In a section of a California tree, a part of which we have seen, more than two thousand such rings were counted, showing that these trees must have lived in the times of David and perhaps of Abraham.

In the earlier ages of the world, some great flood or floods swept over the immense growths of the warmer climes, which then, no doubt, included what is now called Ohio and Pennsylvania. In process of time, this growth was covered with earth and stones, and eventually became "coal," the anthracite and bituminous, with which we are so familiar; and the very identical carbon which the sun light of ages ago separated for the purpose of vegetation, is now, by its combination with its old associate oxygen, returning to its original condition of carbonic acid gas, and in making that change by what we call "burning," warms our houses, lights up our streets, and is preparing to grease our rail cars, by the oil which it is capable of yielding.

